# Combating Dermatologic Misinformation on Social Media via a Training Program for Health Care Professionals: Protocol for a 2-Phase Pilot Mixed Methods Study

**DOI:** 10.2196/81003

**Published:** 2026-04-08

**Authors:** Aliyyat Afolabi, Elijah Brown, Joanne Chen Lyu, Pamela M Ling, Andrea M Rustad, Anna E Davis, Beth L Hoffman, Melissa Pugliano-Mauro

**Affiliations:** 1 School of Medicine, University of Pittsburgh, Pittsburgh, PA, United States; 2 TSET Health Promotion Research Center, Stephenson Cancer Center, University of Oklahoma Health Campus, Oklahoma City, OK, United States; 3 Department of Family and Preventive Medicine, College of Medicine, University of Oklahoma Health Campus, Oklahoma City, OK, United States; 4 Center for Tobacco Control Research and Education and Division of General Internal Medicine, University of California, San Francisco, San Francisco, CA, United States; 5 Department of Dermatology, University of Wisconsin, Madison, WI, United States; 6 Department of Dermatology, University of Pittsburgh Medical Center, Pittsburgh, PA, United States; 7 Department of Behavioral and Community Health Science, University of Pittsburgh School of Public Health, 130 De Soto St, Pittsburgh, PA, 15261, United States, 1 412-624-5859

**Keywords:** dermatology, social media, misinformation, health communication, medical education, TikTok, Instagram, digital literacy

## Abstract

**Background:**

The rise of social media has drastically altered the landscape of health information dissemination. While these platforms can promote health literacy, they are also rife with skin care misinformation, often spread by nonexperts. This has led to harmful consequences, including improper product use and delayed treatment. Despite their potential to mitigate misinformation, dermatologists and health care professionals remain underrepresented on platforms such as TikTok and Instagram.

**Objective:**

This protocol describes a pilot study designed to evaluate the feasibility, acceptability, and preliminary efficacy of a social media training program aimed at equipping dermatology-focused health care professionals and students with skills to create accurate, engaging educational content.

**Methods:**

This sequential mixed methods study will be conducted at the University of Pittsburgh. Phase 1 consists of a 5-module online training program completed by 50 health care professionals and students in dermatology or dermatology-adjacent fields. Pretraining and posttraining surveys will be conducted to assess changes in participants’ knowledge, confidence, intent to engage in public-facing dermatology education, and satisfaction with the training (posttraining survey only). Phase 2 will involve the selection of 12 participants from phase 1 to apply the training by creating and posting short-form dermatologic educational videos on a study-managed social media channel. Exploratory outcomes include social media engagement metrics, qualitative content analysis of public comments, and individual qualitative interviews with participants to assess audience response and potential knowledge impact.

**Results:**

Recruitment for phase 1 of the study is currently underway. As of January 2026, a total of 46 out of the target 50 participants have been successfully enrolled. Data collection for phase 1 is expected to be completed by the end of April 2026. Data analysis will begin shortly thereafter, with final analyses anticipated to be completed by September 2026. Recruitment for phase 2 is expected to begin in April 2026, with the goal of concluding data collection and analysis by the end of 2028.

**Conclusions:**

This pilot study will inform the feasibility and refinement of social media training interventions designed to empower health care professionals to engage in evidence-based dermatologic health communication online.

## Introduction

In today’s digital age, individuals have unprecedented access to skin care information through various online platforms. While this democratization of information has the potential to enhance public knowledge, it has also led to the widespread dissemination of misinformation [1-4]. Social media platforms are inundated with skin care recommendations and quick fixes promoted by individuals lacking formal dermatological training [2,5,6]. One study found that 38% of popular dermatology-related Instagram accounts are managed by health care professionals and just 4% of these are operated by board-certified dermatologists [1,7,8]. This imbalance has facilitated the circulation of unsupported and potentially harmful skin care practices, contributing to widespread misunderstanding of basic dermatological principles and associated negative health outcomes [9]. For example, a case review by Szeto et al [8] that looked at social media in dermatology described patients who adopted harmful skin care practices after encountering inaccurate dermatologic content on social media, including improper chemical peels, excessive exfoliation, and unverified acne treatment [8,10]. These practices resulted in adverse effects such as skin irritation, hyperpigmentation, and worsened acne. Additional research has shown that viral skin care trends on platforms such as TikTok and Instagram have encouraged unsafe behaviors, including the misuse of retinoids, inappropriate chemical exfoliation, and self-diagnosis of conditions [11].

However, when used appropriately, social media offers substantial potential benefits, including interphysician collaboration, patient education, and public health promotion [12]. Prior studies demonstrate that health care professionals’ active engagement on social media can improve public understanding of medical topics and help counter the spread of misinformation [12,13].

Despite this potential, dermatologists remain significantly underrepresented in digital spaces compared with nonexpert influencers, creating a persistent gap in accessible, evidence-based skin care guidance [14].

To address this gap, we developed a series of online social media training modules designed for dermatologists, health care professionals in dermatology-adjacent fields, and health professional students [15]. These modules aim to equip participants with the skills and confidence to create engaging, short-form videos that promote evidence-based dermatologic information. Through instruction in storytelling, content structure, production, and platform-specific optimization strategies, the training empowers participants to actively take part in shaping the digital narrative around dermatologic health. The modules were developed in collaboration with a social media expert and consist of concise instructional videos followed by interactive questions to reinforce learning. The overarching goal is to prepare trusted health care professionals to serve as credible digital voices, counter misinformation, and expand access to accurate dermatologic education through independently created content.

This manuscript describes the development of these training modules and presents the protocol for a 2-phase pilot research study. Phase 1 evaluates the acceptability and preliminary efficacy of the training in improving participants’ knowledge, confidence, and intent to use social media for public dermatologic education. Phase 2 extends this work by examining how selected participants apply the acquired skills to create and disseminate dermatology-focused educational videos on social media platforms. This phase explores video reach and engagement, as well as how the content creation process influences participants’ own confidence and willingness to engage in public health messaging.

While the training program is designed to equip health care professionals and students with practical skills for effective digital communication, the study aims to evaluate the feasibility, acceptability, and preliminary efficacy of the intervention (phase 1) and explore the real-world application of these skills through participant-generated content and audience engagement (phase 2).

## Methods

### Overview

**Figure 1.**
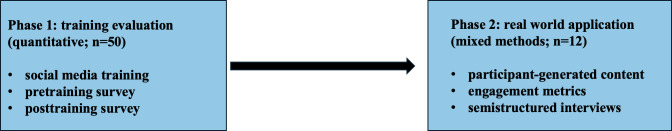
Sequential mixed methods study design. Phase 1 assesses the feasibility, acceptability, and preliminary efficacy of a social media training intervention using pretraining and posttraining surveys. Phase 2 examines the real-world application of training skills using participant-generated content, social media engagement metrics, and semistructured interviews.

This study uses a sequential mixed methods design consisting of 2 interrelated phases (Figure 1). To enhance transparency and reproducibility, reporting of the study design, participant flow, and outcome measures will follow the TIDieR (Template for Intervention Description and Replication) checklist, ensuring detailed description of the intervention content, delivery, and evaluation methods (see Multimedia Appendix 1 for the full checklist).

### Phase 1 Study Design

Phase 1 uses a quantitative, collaborative design for piloting an online training program. The training focuses on teaching physicians and health professional students in dermatology, or fields in which dermatological conditions are commonly seen (eg, pediatrics), how to create engaging social media videos on different popular platforms such as Instagram, TikTok, and YouTube. The study will be conducted at the University of Pittsburgh Medical Center (UPMC) and the University of Pittsburgh. A total of 50 participants will be enrolled in the training program on the Qualtrics platform (Qualtrics International Inc). The modules are hosted on Qualtrics. This platform was selected because it allows for seamless integration of video-based training content with embedded knowledge checks and surveys; supports conditional logic for progressive learning; and enables real-time data collection to evaluate participant engagement, knowledge, and confidence. Qualtrics is also widely used in academic and health care settings, making it well suited for research-based educational interventions. The training consists of 5 short instructional video modules (approximately 8‐12 minutes each), each followed by a brief quiz containing 2 to 3 questions to reinforce learning and ensure engagement. Phase 1 will be focused on evaluating the training’s impact on participants’ confidence and knowledge of and engagement with dermatologic content creation, which will be assessed via pre- and posttraining surveys; the postsurvey will also include items to measure satisfaction with the program (Table 1). A sample size of 50 participants was selected to exceed the recommended minimum of 30 participants for pilot studies assessing questionnaire reliability and preliminary efficacy. This approach accounts for anticipated nonresponse and incomplete data and aligns with published methodological guidance for pilot research as outlined by Bujang et al [16]. Participants who withdraw prior to completing phase 1 will not be replaced, consistent with the exploratory nature of a pilot study. A pretest-posttest single-group design was selected due to the pilot nature of the study and its focus on assessing within-participant changes following the intervention. Outcomes including knowledge, confidence, and intent to engage in digital dermatologic education were selected as proximal indicators of participants’ readiness to apply training skills.

**Table 1.** Phase 1 measures and outcomes assessed in the pre- and posttraining surveys.

Domain	Measure type	Item or description	Scale or response format	Time point
Demographics	Self-report	Age, ethnicity, educational level, student or professional status, field role, and experience	Multiple choice	Baseline
Prior experience	Self-report	“Have you ever created or been involved in creating educational content on social media?”	“Yes,” “No,” or “Prefer not to say”	Baseline
Knowledge	Quiz	10 multiple-choice questions on video structure, pacing, ethics, and engagement, among other topics	Multiple choice (correct or incorrect)	Baseline and after the training
Satisfaction	Self-report	Satisfaction with the information presented, ability to complete the modules independently, time required, and overall experience	7-point Likert scale (1=“completely dissatisfied”; 7=“completely satisfied”)	After the training

### Phase 2 Study Design

Following completion of phase 1, a total of 12 participants will be selected to take part in phase 2 to demonstrate the application of training skills and explore the real-world dissemination of dermatologic educational content. Purposive sampling will be used to ensure diversity in training level, professional background, and engagement with the training modules. The final sample size of 12 participants was determined based on our goal of achieving thematic saturation with individual qualitative interviews as qualitative research guidance indicates that 9 to 17 interviews are typically sufficient to achieve thematic saturation [17].

In this phase, participants will be asked to create at least 3 short-form educational videos over a 1-month period. These videos will be posted to our study-managed social media accounts (Instagram, TikTok, and YouTube) under the Pitt channel, with the goal of correcting common skin care misconceptions using the principles taught in the training modules.

Participants will generate their own scripts using reliable dermatologic information sourced from the American Academy of Dermatology’s “Stats and facts” web page as it is considered a trusted source by board-certified dermatologists and is affiliated with the world’s largest dermatology organization [18]. All video scripts will be reviewed by the principal investigator, who is also a board-certified dermatologist and Mohs surgeon, in addition to the coauthor, who is a dermatology resident, to ensure medical accuracy prior to posting. Rather than assigning specific pieces of misinformation to rebut, participants will identify commonly encountered dermatologic myths or knowledge gaps based on their clinical experience and training. They will then create original, engaging short-form videos to educate the public and improve dermatologic health literacy.

To assess digital impact, we will collect quantitative engagement metrics for each video, including likes, comments, shares, saves, views, and engagement rate ([likes + comments + shares + saves]/views) [19]. Because the Pitt study channel is newly created, we expect the initial benchmarks to be zero, and growth over time will serve as an exploratory indicator of how participants applied the training intervention in a real-world digital environment.

We will conduct qualitative content analysis of the videos created and the public comments left on these videos to evaluate how participants select their topics, present themselves, and use techniques to create engaging content. We will also assess how closely the videos align with the training guidance, as well as audience sentiment, perceived credibility, and engagement with dermatologic concepts. This qualitative analysis will complement the quantitative metrics and provide deeper insights into audience knowledge and attitudes in response to the videos.

Finally, we will conduct individual qualitative interviews with all 12 participants to explore their content creation experience, perceived barriers to digital health communication, confidence in public engagement, and perceived benefits of participating in this intervention. Interviews will be conducted by trained research assistants using a semistructured interview guide.

### Outcome Measures

For phase 1, the primary outcomes are feasibility, acceptability, and preliminary efficacy of the social media training modules. Feasibility will be assessed using module completion rates and participant retention from the pretraining to posttraining surveys. Acceptability will be evaluated using posttraining survey items on satisfaction with the information presented, ability to complete the modules independently, and perceived relevance of the material using a 7-point Likert scale (1=“completely dissatisfied”; 7=“completely satisfied”). Preliminary efficacy will be assessed through knowledge gain on 10 multiple-choice questions aligned with the training content, as well as participant-reported confidence and intent to engage in digital dermatologic education. Exploratory measures include participant engagement with each module, such as quiz completion and time spent on each module.

For phase 2, outcomes focus on the real-world application of training skills and the potential digital impact of participant-generated content. Quantitative engagement metrics for each video posted to the study-managed social media accounts (Instagram, TikTok, and YouTube) will include views, likes, shares, comments, saves, and engagement rate ([likes + comments + shares]/views). Content fidelity will be assessed by comparing participant videos to the training guidance, with all scripts reviewed for medical accuracy by study investigators. Qualitative outcomes will be collected through semistructured interviews exploring participants’ experiences with content creation, perceived barriers and facilitators, and confidence in public health communication. Additionally, a qualitative content analysis of public comments on the videos will assess audience engagement, sentiment, perceived credibility, and evidence of knowledge uptake or correction of misinformation. Viewer-level changes in knowledge, attitudes, or behavior are beyond the scope of this pilot study but will be considered in future research.

### Participants and Inclusion and Exclusion Criteria

For phase 1, a total of 50 participants from the University of Pittsburgh and UPMC will be enrolled in the study. Participants will be eligible if they are physicians (MD or DO), physician associates (PA), clinical registered nurses (RN and BSN), or nurse practitioners (NP) in dermatology or dermatology-adjacent fields such as pediatrics, rheumatology, family medicine, and allergy or students pursuing degrees in these fields (eg, medical, nursing, and physician associates) interested in dermatology or dermatology-adjacent fields.

Exclusion criteria include health students without an expressed interest in dermatology and health professionals whose patient population with dermatological conditions is rare (eg, general surgery, orthopedics, or psychiatry).

Interest in dermatology will be assessed via participant self-report. We will use the following single categorical question to assess professional background and interest in dermatology: “Are you currently a student interested in pursuing a career in the field of dermatology or a health professional working in the field of dermatology or a related health field?” Responses will include four options: (1) student, (2) working in the field or field-related (ie, conducting dermatological research), (3) both (student and working in the field), and (4) neither. Participants selecting options 1, 2, or 3 will be required to specify their role. Students in medical, nursing, or physician associate programs and health care professionals with MD, DO, PA, or NP degrees in rheumatology, allergy, pediatrics, and family medicine will be included in the study.

For phase 2, participants will be eligible if they have completed phase 1 of the study and have access to a smartphone to create videos. If requested, study staff will provide a tripod (valued at US $20) to support video creation.

### Recruitment

A multimodal approach will be used to recruit participants for phase 1. Medical student recruitment will be facilitated through collaboration with the University of Pittsburgh Office of Medical Education using direct communication channels (emails and SMS text messages) to ensure comprehensive outreach across academic years, specialty interests, and backgrounds. Recruitment will be coordinated with the health sciences library, which will distribute mass email invitations detailing study objectives, eligibility criteria, and instructions for participation. To recruit practicing health professionals, informational flyers will be distributed at UPMC Children’s Hospital of Pittsburgh and adult dermatology clinics affiliated with UPMC. These sites have been selected for their high patient and clinician traffic, allowing for targeted outreach to relevant health care providers.

For phase 2, participants from phase 1 will be sent an email containing a flyer with details about phase 2 of the study. From those who express interest, a purposive sampling approach will be used to select 12 individuals to ensure diversity in training level, professional background, and engagement with the training modules. Selection will be based on expressed interest and diversity considerations and will not be based on quiz performance, response time, or other performance-based metrics. Selected participants will then be contacted via email with a formal invitation to take part in phase 2, along with a consent form outlining expectations, time commitment, and next steps.

### Module Development

Each module was developed by the primary author, who has experience in creating health-related content for social media platforms, and was reviewed by the principal investigators. Prior to development, the first author conducted a content analysis of high-performing videos across TikTok, Instagram, and YouTube to identify strategies for effective audience engagement, including storytelling techniques, visual presentation, and commonly used production equipment. These insights, combined with personal experience in content creation, informed the scripting and design of the modules to ensure that they were concise, engaging, and appropriate for social media dissemination.

The intervention consists of 5 short, interactive modules designed to empower students and health professionals to create engaging and culturally sensitive short-form videos for social media. These modules are led by a medical student with an interest in dermatology and experience in communication and content creation. Each module builds on the last to provide a comprehensive foundation in video creation, health storytelling, ethical considerations, and platform-specific optimization.

**Figure 2.**
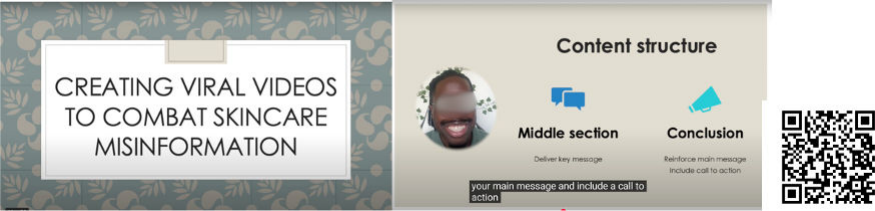
Screenshot of one of the modules, titled “Creating Viral Videos to Combat Skincare Misinformation.” A QR code is included for direct access to the video module.

The first module, “Fundamentals of Short-Form Video Creation,” introduces participants to the core principles of effective short-form videos. It covers content structure (opening, message delivery, and call to action); pacing techniques to retain viewer attention; and strategies for audience engagement, such as using prompts and captions. Visual examples are included to demonstrate each concept (Figure 2). The second module, “Storytelling Techniques for Dermatology,” emphasizes the role of narrative in health communication. Participants learn how to use relatable patient stories, show treatment journeys, highlight universal themes (eg, empowerment), and end videos with actionable health messages. The importance of authenticity and obtaining patient consent when using real testimonials is also covered.

The third module, “Video Production Tools and Techniques,” provides an overview of essential production elements, including lighting, sound, editing software, and camera composition. Participants are introduced to accessible tools (eg, video editing, ring lights, and lavalier mics) and guided through the video editing process from raw footage to finalized content.

The fourth module, “Ethical and Culturally Sensitive Practices,” addresses ethical video creation with a focus on diversity, inclusion, and respect for cultural practices. Topics include using inclusive language, avoiding stereotypes, honoring traditional practices, obtaining informed consent, and using content to highlight disparities in dermatologic care.

The last module, “Optimizing Videos for Social Media Platforms,” guides participants in tailoring content for specific platforms: Instagram, TikTok, and YouTube. It explores best practices for video formatting, captioning, trends, hashtags, and audience targeting. Participants learn how to adapt content tone, length, and structure for each platform to maximize reach and impact.

After each video, a quiz will be administered to ensure that participants actively engage with and retain the material.

### Statistical Analysis

All quantitative analyses will be conducted using SPSS Statistics (version 26; IBM Corp). Survey datasets will be screened for missing data and outliers prior to analysis. Descriptive statistics (counts, percentages, means, and SDs) will be calculated for participant demographics, module completion, retention, and satisfaction metrics.

For phase 1, pre- and posttraining survey knowledge items will be analyzed using the McNemar test to evaluate within-participant changes. Likert-scale items assessing satisfaction and perceived relevance will be summarized using frequencies and measures of central tendency. A significance threshold of a *P* value below .05 will be applied for all quantitative analyses.

For phase 2, social media engagement metrics (views, likes, shares, saves, and comments) will be summarized using means, medians, and engagement ratios as exploratory indicators of public reach and receptivity.

### Qualitative Analysis

Qualitative data will be managed and analyzed using the NVivo software (Lumivero). Public social media comments will undergo qualitative content analysis, with coding for evidence of knowledge uptake, misinformation correction, curiosity, and trust in dermatologic guidance.

Phase 2 interviews will be audio recorded, transcribed verbatim, and analyzed using thematic analysis following Braun and Clarke’s 6-phase framework [20]. Two independent coders will familiarize themselves with the transcripts, generate initial codes aligned with the study aims, review and refine themes, and resolve discrepancies through consensus. Recruitment and analysis will continue until thematic saturation is achieved.

### Ethical Considerations

This study was reviewed and approved by the University of Pittsburgh Office of Research Protections (approval STUDY24070132). The study was determined to involve minimal risk to participants.

All participants will receive detailed information about the study’s aims, procedures, potential risks, and benefits and will provide informed consent electronically prior to participation, ensuring voluntary and fully informed participation in the study. Participants may withdraw at any time without penalty. No personally identifiable information will be collected beyond what is necessary for study purposes. All data will be stored on secure, password-protected institutional servers and will be accessible only to authorized members of the research team. As a thank-you for their time, participants will receive a US $10 gift card upon completion of phase 1. Those selected for and who complete phase 2 will receive a US $50 gift card.

The study will be conducted in accordance with institutional guidelines and applicable ethical standards for research involving human participants.

## Results

Recruitment for phase 1 of the study is currently underway. As of January 2026, a total of 46 out of the target 50 participants have been successfully enrolled. Data collection for phase 1 is expected to be completed by the end of April 2026. Data analysis will begin shortly thereafter, with final analyses anticipated to be completed by September 2026. Recruitment for phase 2 is expected to begin in April 2026, with the goal of concluding data collection and analysis by the end of 2028 and publishing results in spring 2029.

## Discussion

### Expected Findings

This study is among the first to explore an online social media training program designed to equip health professional students and health care providers in dermatology or dermatology-adjacent fields with the practical skills to create engaging, evidence-based digital content. As social media platforms increasingly serve as the primary sources of health information, particularly for younger populations, ensuring the availability of accurate and accessible dermatologic content is increasingly urgent. Due to its pilot nature, this study is not designed to establish causal effects on public knowledge, attitudes, or behavior but, rather, to inform the feasibility and refinement of future interventions.

Although this pilot does not directly measure the causal impact of participant-created videos on viewer outcome, it is grounded on the premise that strengthening dermatology professionals’ digital communication skills may increase their confidence and willingness to engage in public-facing education. Over time, this engagement has the potential to contribute to a broader and more sustained presence of accurate dermatologic messaging in the digital space [21]. By training both current and future health care providers to create engaging, evidence-based content, this intervention addresses a critical educational gap while empowering professionals to actively shape the online narrative around skin health [22]. The inclusion of core training components, such as storytelling, ethical content development, and platform-specific optimization, ensures that participants are equipped with both the technical and strategic tools necessary for meaningful digital engagement.

These goals align with recent findings indicating that health care professionals increasingly view social media as a valuable educational tool despite persistent concerns about privacy and misinformation. In a national survey, most dermatologists in the United States reported a willingness to increase their online engagement if better training and institutional support were available [23]. Similarly, structured digital media training has been shown to translate into real-world practice, with nearly 60% of trained providers applying their new skills to create patient-facing content [24].

Phase 2 of the study addresses the potential impact of participant-generated videos through a combination of engagement metrics (eg, likes, shares, and comments), qualitative analysis of viewer comments, and in-depth interviews with participants. Comment analysis will explore indicators such as expressions of learning, curiosity, misinformation correction, and attitudinal shifts. Participants will also reflect on perceived audience reactions and whether their content generated meaningful interactions.

The second phase—focused on video creation and performance metrics—adds a critical dimension by evaluating how educational content performs in real-world digital environments. This allows for preliminary insight into the feasibility of scaling such interventions and highlights the potential for trained health professionals to serve as trusted voices within an oversaturated media landscape. Metrics such as likes, views, shares, and comments will be used as proxies for audience receptivity and public interest in evidence-based dermatologic content. Participant reflections will also help identify key facilitators of and barriers to digital engagement, which will be essential for designing future interventions. While combating misinformation is a central goal, this study also aims to build long-term digital communication capacity by training health care professionals to confidently and ethically engage diverse online audiences.

The study is not without limitations. As a pilot study with a relatively small sample size for the content creation phase (n=12), findings on engagement and reach will be exploratory in nature. Additionally, participant motivation and prior familiarity with social media may influence both the quality of the content and its performance. Future studies may consider stratifying participants based on baseline digital literacy and including a control group for more robust comparisons.

Despite these limitations, this protocol lays the foundation for a scalable, educationally grounded approach to addressing the growing challenge of dermatologic misinformation online [25]. If successful, this model could be replicated across other medical disciplines and programs and used to cultivate a new generation of digitally fluent, public-facing health professionals.

### Conclusions

This pilot study aims to address the growing challenge of dermatologic misinformation on social media by equipping health professional students and providers with the tools and confidence to create accurate, engaging, and accessible educational content. Through a structured training program and real-world application phase, the study will evaluate both knowledge gains and the feasibility of digital content dissemination. By empowering participants to contribute to evidence-based discourse online, this intervention has the potential to enhance public dermatologic literacy; promote digital health equity; and serve as a model for scalable, specialty-specific training across health professions.

## Supplementary material

10.2196/81003 Multimedia Appendix 1 TIDieR (Template for Intervention Description and Replication) checklist.

## Abbreviations

TIDieR: Template for Intervention Description and Replication
UPMC: University of Pittsburgh Medical Center
